# Impact of an In-Hospital postoperative imaging after uncomplicated elective posterior lumbar fixation: A case series

**DOI:** 10.1007/s10143-024-03055-y

**Published:** 2024-10-30

**Authors:** Mohamed AR AbdelFatah, Abdelrahman El Gayar, Mostafa K. Ghobashy, Sameh Hefny

**Affiliations:** Neurosurgery Department, Faculty of Medicine, Ain Shams University, Cairo, Egypt

**Keywords:** Case series, Degenerative lumbar spine, Lumbar fixation, Routine imaging

## Abstract

Purpose: Following spinal fixation, postoperative imaging is routinely performed. The value of routine postoperative imaging and its impact on the surgical decision remains uncertain, especially in degenerative cases. Moreover, routine postoperative imaging is not free and is an ionizing radiation. This study investigated the value of postoperative imaging after uneventful uncomplicated elective posterior lumbar fixation. Methods: This case series retrospectively reviewed the medical records of patients who underwent elective posterior lumbar fixation surgeries at our institution within two years. A series of 98 cases met our selection criteria. Their mean age was 51.2 years. We reviewed the decisions taken after performing the routine postoperative images. We searched for further diagnostic or imaging studies, revision surgery, or an extended hospital stay. Results: We found no particular decision was made after performing the postoperative imaging after uneventful uncomplicated elective posterior lumbar fixation surgeries. Moreover, there was no change in the hospital stay or the regular postoperative clinical management for all the included patients. No revision surgery was required based on the postoperative routine images. Conclusions: We found that routine postoperative imaging after posterior fixation of a degenerative lumbar spine is of limited value. A randomized, controlled study is helpful to confirm this finding.

## Introduction

Degenerative lumbar spine is the most common indication for elective lumbar fixation surgeries [1]. Pedicle screw fixation is the preferred method of posterior lumbar fixation surgery. This method provides three-column support for the vertebrae, contributing to the biomechanical strength of the construct [2]. Pedicle screws are often inserted based on anatomical landmarks, assisted by intraoperative fluoroscopy [3].

Postoperative nonspecific symptoms like low back pain and non-radicular pain are frequently observed [4]. Following spinal fixation, postoperative imaging is routinely performed. Such routine imaging may include X-rays in different positions or computed tomography (CT) with sagittal and coronal reconstruction [5].

To our knowledge, there is no standard of care regarding early postoperative imaging after spinal fusion. The value of routine postoperative imaging and its impact on the surgical decision remains uncertain, especially in degenerative cases. Moreover, routine postoperative imaging is not free and is ionizing radiation.

This study investigated the value of routine postoperative imaging after posterior fixation of a degenerative lumbar spine.

## Materials and methods

This study is a retrospective case series. We reported this case series following the PROCESS Guidelines [6]. We conducted the study procedures following the Ethical Guidelines for Medical and Health Research Involving Human Subjects and its later amendments (the Declaration of Helsinki). The Research Ethics Committee of the Faculty of Medicine at Ain Shams University (FWA 000017585) approved this study. Informed consent was obtained as required.

We reviewed the medical records from July 1, 2022, to July 1, 2024, at a single tertiary hospital (our university hospital).

### Inclusion criteria

- Elective posterior lumbar fixation for a degenerative lumbar disease.
- Uneventful uncomplicated surgeries.

### Exclusion criteria (postoperative imaging was clinically indicated)

- Postoperatively unimproved or residual radicular pain.
- Postoperatively new neurological deficit or new radicular pain.
- Postoperative CSF leaks.

Ninety-eight cases met the selection criteria for this study. We collected the following data: patients’ demographics; preoperative diagnosis and clinical state; operative notes; postoperative clinical and radiological findings; and postoperative management, including the decisions taken after performing the postoperative images. We searched for further diagnostic or imaging studies, revision surgery, or an extended hospital stay.

Surgeries were performed in a standardized fashion, consisting of midline skin incision, bilateral muscle separation, placement of pedicular screws assisted with intraoperative imaging fluoroscopy in both AP and lateral views, followed by decompression of the central canal and the lateral recesses, and then partial unroofing of the nerve roots at their foraminal entry point. The wound was closed in layers. A subfascial drain was routinely placed.

Postoperatively, the patients were allowed to walk on the day of the operation. The subfascial drain was routinely removed on the first postoperative day unless there was excess blood > 50 ml per 12 h or a cerebrospinal fluid (CSF) leak. On the second postoperative day, a plain X-ray of the lumbosacral spine in various views or a CT lumbosacral spine with sagittal and coronal reconstruction was routinely performed. *The choice of X-ray versus CT depended on the surgeon’s preference.*

Routine imaging is defined as any imaging obtained in the absence of any clinical indication. The patients were routinely discharged on the morning of the third postoperative day. The follow-up in the outpatient clinic was every two weeks in the first month and then monthly after that for three months.

### Statistical analysis

Data was collected and entered into the Statistical Package for Social Science (IBM SPSS) version 23. We described the quantitative data by the mean and range, and the qualitative data by frequencies.

## Results

A hundred and sixty-two cases met our inclusion criteria; however, we excluded sixty-four according to the exclusion criteria. As a result, a series of 98 cases were eligible for this study. The average age of the included patients was 51.2 years (ranging from 34 to 67 years).

Table 1 shows the preoperative clinical characteristics of the included patients. Table 2 summarizes the preoperative diagnosis. The indication for fixation for multilevel disc prolapse, lumbar canal stenosis, and recurrent disc prolapse was based on the surgeon’s decision.

**Table 1:** The preoperative clinical characteristics

Preoperative clinical characteristics	Number of patients (%)
**Gender**	
Male	45 (45.9%)
Female	53 (54.1%)
**Medical diseases**	
Controlled hypertension	24 (24.4%)
Controlled diabetes mellitus	17 (17.3%)
Ischemic heart disease	3 (3%)
Obesity (Body mass index ≥ 30)	46 (46.9%)
Smoking	36 (36.7%)
Chronic liver disease	4 (4%)
**Preoperative neurologic deficit**	
Partial foot drop	9 (9.1%)
Weak dorsiflexion of big toe	6 (6.1%)
Weak foot plantar flexion	3 (3%)
**Preoperative symptoms**	
Low back pain	84 (85.7%)
Radiculopathy	77 (78.5%)
Claudication	21 (21.4%)

**Table 2:** The preoperative diagnosis of the included patients

Preoperative diagnosis	Fused Levels	*N* (%)
Recurrent disc prolapse	L3-4 L4-5 L5-S1	3 (3%) 5 (5.1%) 1 (1%)
Degenerative spondylolisthesis	L3-4 L4-5 L5-S1	12 (12.2%) 24 (24.4%) 4 (4%)
Isthmic spondylolisthesis	L4-5 L5-S1	11 (11.2%) 6 (6.1%)
Multiple disc prolapse	L2-3-4-5	4 (4%)
Lumbar spinal canal stenosis	L3-4 L4-5	8 (8.1%) 9 (9.1%)
Adjacent segment disease	L2-3-4-5 L3-4-5-S1 L4-5-S1	4 (4%) 2 (2%) 5 (5.1%)

For all of the included patients, there were no reported intraoperative complications. Postoperatively, all the patients experienced improvement in their radicular pain. There was no incidence of new radicular symptoms or new neurological deficits. The subfascial drain was removed on the first postoperative day. There was no reported CSF or excess blood in the drain.

On the second postoperative day, routine imaging of the lumbosacral spine was performed. A routine plain X-ray of the lumbosacral spine in various views was ordered for 42 patients. A CT lumbosacral spine with sagittal and coronal reconstruction was ordered for 56 patients. *The choice of X-ray versus CT depended on the surgeon’s preference and was unrelated to the pathology or surgery.*

No further imaging studies or revision surgery was ordered after performing the routine postoperative imaging. All the patients were discharged on the morning of the third postoperative day.

## Discussion

Currently, many spine surgeons order routine in-hospital postoperative imaging after lumbar fixation [7, 8]. The impact of these routine images on the surgical decision-making process remains uncertain.

This study retrospectively investigated the value of postoperative imaging after uncomplicated elective posterior lumbar fixation surgeries. We emphasize that postoperative spinal imaging is clinically indicated for patients who experience a postoperative neurological deficit or new radicular pain. In addition, immediate postoperative imaging is necessary after a complicated surgery or repeated screw malposition.

According to our study aim, we had to exclude all the patients who underwent non-routine postoperative imaging, like patients who experienced a postoperative new neurological deficit or new radicular pain. We found from our study that no particular decision was taken after performing *the routine* postoperative imaging, nor was there a change in the hospital stay. In addition, no revision surgery was required based on the postoperative *routine images*. Similarly, Molinari et al. (2012) and Ronald et al. (2021) did not find great value in routine postoperative imaging after degenerative spinal fusion [9, 10].

Although the decision of fixation is debatable in multilevel disc prolapse, lumbar canal stenosis, and recurrent disc prolapse, discussing this debate is out of the scope of this paper. The intraoperative fluoroscopy allows verification of the position of the placed hardware. In addition, it can exclude the presence of residual gauze.

Spine surgeons traditionally order postoperative imaging to document surgical success, to reassure patients, and for medicolegal aspects [11]. However, surgeons could keep a digital copy of the intraoperative images. In addition, there was no evidence to support any detectable movement of the spinal instrumentation in the immediate postoperative period for degenerative cases [9].

Routine postoperative spinal imaging may be of clinical significance after the fixation of traumatic and pathological fractures because of their greater propensity for postoperative instability [9].

Spinal fixation represents one of the most commonly performed surgical procedures. Hence, routine postoperative images may greatly burden any country’s resources, especially the developing ones [7]. Furthermore, exposure to ionizing radiation (X-ray or CT) without a clinical indication is hazardous and may be unethical [12, 13]. Moreover, routine investigations, which are unlikely to alter the clinical decision, constitute a psychological burden on the patients and result in the misuse of the hospital staff, including nurses, porters, technicians, and radiologists.

We recommend keeping a digital copy of all spinal instrumentation’s intraoperative final X-rays. Interestingly, Molinari et al. (2012) concluded that the intraoperative fluoroscopic image is an accurate and cost-effective baseline study after a single-level fusion of a degenerative spine [9]. In addition, we recommend that postoperative imaging should not be routine and should be individualized based on the patient’s clinical condition. A prospective, randomized, controlled study is needed to strengthen this recommendation.

Wide discussion in different spinal societies is necessary to produce definitive clinical practice guidelines to avoid unnecessary and potentially harmful postoperative images.

The limitations of this case series are the single-institutional experience and the retrospective nature. Furthermore, our study was limited to patients with degenerative spinal diseases fused with pedicle screws. This potential selection bias may limit the generalizability of our findings to other patients undergoing lumbar fusion surgeries.

## Conclusion

This study found that postoperative imaging after uncomplicated posterior fixation of a degenerative lumbar spine may be of limited value for the patients as no specific clinical decision was made after performing the routine images. A randomized, controlled study is helpful to confirm this finding. It is recommended that the postoperative images not be routine but should be individualized based on the patient’s clinical condition.

## Data Availability

No datasets were generated or analysed during the current study.
